# Cellular and molecular mechanisms of cigarette smoke-induced lung damage and prevention by vitamin C

**DOI:** 10.1186/1476-9255-5-21

**Published:** 2008-11-11

**Authors:** Shuvojit Banerjee, Ranajoy Chattopadhyay, Arunava Ghosh, Hemanta Koley, Koustubh Panda, Siddhartha Roy, Dhrubajyoti Chattopadhyay, Indu B Chatterjee

**Affiliations:** 1 Dr. B. C. Guha Centre for Genetic Engineering and Biotechnology, University College of Science, Kolkata 700019, India; 2 Sealy Center for Molecular Science, University of Texas Medical Branch, Galveston, Texas 77555-1079, USA; 3 National Institute of Cholera and Enteric Diseases, P33, CIT Road, Kolkata 700010, India; 4 Indian Institute of Chemical Biology, 4, Raja S. C. Mullick Road, Kolkata 700032, India

## Abstract

**Background:**

Cigarette smoke-induced cellular and molecular mechanisms of lung injury are not clear. Cigarette smoke is a complex mixture containing long-lived radicals, including p-benzosemiquinone that causes oxidative damage. Earlier we had reported that oxidative protein damage is an initial event in smoke-induced lung injury. Considering that p-benzosemiquinone may be a causative factor of lung injury, we have isolated p-benzosemiquinone and compared its pathophysiological effects with cigarette smoke. Since vitamin C is a strong antioxidant, we have also determined the modulatory effect of vitamin C for preventing the pathophysiological events.

**Methods:**

Vitamin C-restricted guinea pigs were exposed to cigarette smoke (5 cigarettes/day; 2 puffs/cigarette) for 21 days with and without supplementation of 15 mg vitamin C/guinea pig/day. Oxidative damage, apoptosis and lung injury were assessed *in vitro*, *ex vivo *in A549 cells as well as *in vivo *in guinea pigs. Inflammation was measured by neutrophilia in BALF. p-Benzosemiquinone was isolated from freshly prepared aqueous extract of cigarette smoke and characterized by various physico-chemical methods, including mass, NMR and ESR spectroscopy. p-Benzosemiquinone-induced lung damage was examined by intratracheal instillation in guinea pigs. Lung damage was measured by increased air spaces, as evidenced by histology and morphometric analysis. Oxidative protein damage, MMPs, VEGF and VEGFR2 were measured by western blot analysis, and formation of Michael adducts using MALDI-TOF-MS. Apoptosis was evidenced by TUNEL assay, activation of caspase 3, degradation of PARP and increased Bax/Bcl-2 ratio using immunoblot analysis and confocal microscopy.

**Results:**

Exposure of guinea pigs to cigarette smoke resulted in progressive protein damage, inflammation, apoptosis and lung injury up to 21 days of the experimental period. Administration of 15 mg of vitamin C/guinea pig/day prevented all these pathophysiological effects. p-Benzosemiquinone mimicked cigarette smoke in causing protein modification and apoptosis *in vitro *and in A549 cells *ex vivo *as well as apoptosis and lung damage *in vivo*. All these pathophysiological events were also prevented by vitamin C.

**Conclusion:**

p-Benzosemiquinone appears to be a major causative factor of cigarette smoke-induced oxidative protein damage that leads to apoptosis and lung injury. The pathophysiological events are prevented by a moderately large dose of vitamin C.

## Background

Emphysematous lung damage is a prominent component of Chronic Obstructive Pulmonary Disease (COPD), which is a major and growing cause of morbidity and mortality worldwide. Cigarette smoking is by far the most common cause of emphysematous lung damage. It has been hypothesized that excessive proteolysis, lung cell apoptosis and oxidative stress interact as means by which the lung is destroyed in emphysema [1]. Recently the role of apoptosis in pulmonary emphysema has been highlighted [2]. However, the cellular and molecular mechanisms of the pathophysiology of emphysematous lung damage remain enigmatic. This is particularly because cigarette smoke (CS) is a highly complex mixture containing about 4000 compounds, including free radicals and long-lived radicals [3-5]. Long-lived radical(s) present in aqueous extract of CS is tentatively assigned to semiquinone(s) that is cytotoxic and causes protein and DNA damage [4,5]. DNA fragmentation and protein damage are the hallmarks of emphysema [1]. Although the semiquinone(s) present in CS was tentatively identified as p-benzosemiquinone (p-BSQ), this was not isolated. It is yet to be known whether p-BSQ of CS causes apoptosis and emphysematous lung damage. We have addressed this question for better understanding of the cellular and molecular mechanisms of emphysema, so that effective therapeutic strategies could be developed for the prevention of this disease. We have isolated p-BSQ from freshly prepared aqueous extract of CS (AECS) and characterized it. Using various *in vitro*, *ex vivo *and *in vivo *approaches, here we show that p-BSQ largely mimics AECS in causing oxidative protein damage, proteolysis, apoptosis and lung injury in guinea pigs.

Using a guinea pig model developed in our laboratory, we had hypothesized that the sequence of pathophysiological events leading to CS-induced lung injury might be oxidative protein damage, followed by inflammation and apoptosis [6]. So we considered that once protein oxidation was prevented, the subsequent events of apoptosis and lung damage might also be prevented. Previously we had shown that exposure of guinea pigs to cigarette smoke for 7 days causes significant lung injury and that administration of the antioxidant black tea prevents the lung lesions. But the amount of black tea needed was high (about 1 g/kg body weight). The health effect of high consumption of black tea in humans is yet to be known. Earlier we had shown that vitamin C prevents cigarette smoke-induced oxidative protein damage and subsequent proteolysis [7,8]. Moreover, population surveys have linked a low dietary intake of vitamin C with worse lung function [9,10]. Vitamin C is the most common nontoxic essential dietary antioxidant. Here we demonstrate that exposure of guinea pigs to CS for 21 days results in progressive protein damage, apoptosis and lung injury and that administration of a moderately large dose of vitamin C almost completely prevents protein damage, apoptosis and the lung injury. The advantage of using the guinea pig for investigation of emphysema is that like humans it is not only incapable of synthesizing vitamin C [11,12], but also it has anatomical and CS-induced pathophysiological similarities to human [13]. N-acetylcysteine (NAC) is a known antioxidant and there are controversial reports that NAC reduces the severity of COPD [14-16]. So we have also examined the effect of NAC in CS-induced lung damage in our guinea pig model.

## Methods

### Chemicals and reagents

Antibodies against vascular endothelial growth factor receptor (VEGFR2), Bax, Bcl-2, cytochrome c, caspase 3 and anti-mouse-HRP, anti-rabbit HRP antibodies as well as the chemiluminescent kit for immunoblot analysis were obtained from Cell Signaling Technology, Inc. USA. Oxyblot protein oxidation detection kit was obtained from Intergen Company, USA. Antibody of VEGF, MMP-9, MMP-12 and anti-tubulin antibody was obtained from Santa Cruz Biotechnology, Inc, USA. Tetramethyl Rhodamine iso-thiocyanate (TRITC)-conjugated goat anti rabbit antibody and TRITC-conjugated goat anti mouse antibody was purchased from Bangalore Genei (India). The *in situ *cell death detection kit was obtained from Roche. USA. Kit for protein estimation was obtained from Bio-Rad, USA. Vitamin C (99%) was purchased from Merck. All other chemicals were of analytical grade.

### Exposure of Guinea Pigs to Cigarette Smoke (CS)

Male short hair guinea pigs weighing 350–450 g were used for all experiments. All animal treatment procedures met the NIH guidelines [17] and Institutional Animal Ethics committee guidelines. The guinea pigs were fed vitamin C-free diet for 7 days to minimize the vitamin C level of tissues [6,8]. This is because vitamin C is a potential inhibitor of CS-induced protein oxidation [7,8], which would otherwise counteract the damaging effect of CS. The vitamin C-free diet given to the guinea pigs was similar to that described before [18], except that wheat flour was replaced by wheat bran. In brief, the diet was composed of 70% wheat bran, 20% vitamin C-free casein, 8% sucrose, 1% USP XVII salt mixture and 1% AOAC vitamin mixture. After 7 days of vitamin C deprivation, the guinea pigs were subjected to cigarette smoke exposure from 5 cigarettes/animal/day in a smoke chamber, along with supplementation of vitamin C (1 mg, 5 mg or 15 mg/animal/day), as indicated in the respective experiments. Deprivation of vitamin C after 7 days was discontinued to avoid onset of scurvy. One mg of vitamin C per day is approximately the minimum dose needed to prevent scurvy in the guinea pig [6]. An Indian commercial filter-tipped cigarette (74 mm) with a tar content of 20 mg and nicotine content of 1 mg was used throughout the study. The smoke chamber was similar to that of a vacuum desiccator with an open tube at the top and a side tube fitted with a stopcock, as described before [6]. The volume of the chamber was 2.5 litre. The cigarette placed at the top was lit and CS was introduced into the chamber containing the guinea pig by applying a mild suction of 4 cm water through the side tube for 5 sec. Thereafter, the vacuum was turned off and the guinea pig was further exposed to the accumulated smoke for another 40 sec. The total duration of exposure to smoke from one puff was thus 45 sec. The amount of suspended particle per puff was approximately 10 mg. Altogether 2 puffs per cigarette was given, allowing the animal 1 min rest in smoke-free atmosphere to breathe air between each puff. The gap between one cigarette and the next was 1 hour. Pair-fed sham controls were subjected to air exposure instead of CS under similar conditions.

The guinea pigs were divided into the following experimental groups (*n *= 4/group): (i) exposed to air and supplemented with 1 mg or 15 mg vitamin C/animal/day (Air + vit C 1 mg, Air + vit C 15 mg, respectively); (ii) exposed to CS and supplemented with 1 mg or 15 mg vitamin C (CS + vit C 1 mg, CS + vit C 15 mg, respectively). After feeding vitamin C-free diet for 7 days following exposure to air or CS up to 21 days with supplementation of 1 mg/15 mg vitamin C/day, both the sham controls and the CS-exposed guinea pigs were deprived of food overnight and sacrificed next day by diethyl ether inhalation. The lungs were then excised immediately and processed for analysis.

### Isolation of broncho-alveolar lavage fluid (BALF)

After deprivation of vitamin C for 7 days followed by CS exposure for 14 days with supplementation of 1 mg vitamin C/day, three guinea pigs were used separately for the isolation of broncho-alveolar lavage fluid (BALF). After sedation with ketamin (100 mg/kg), the trachea was opened, 6 ml of PBS introduced by a syringe and 3 ml of BALF collected by the same syringe. The collected BALF was centrifuged at 1000 rpm (98 g) for 5 min at 4°C. The residue containing the cells was dispersed in 150 μl of phosphate buffered saline (PBS). A portion of the dispersion was used for cell counting. After proper dilution (about 5 times), cells were counted in a haemocytometer. Neutrophils were counted by light microscopy using Leishman's stain after preparing a smear. The proteins of the supernatant were precipitated with 80% cold acetone and the acetone-free residue was dissolved in 200 μl of lysis buffer containing protease inhibitor cocktail mixture (1×, Roche Applied Science). Protease inhibitor was used to inhibit CS-induced proteolysis of BALF protein. Thirty μg protein equivalent of this solution was subjected to western blot analysis for measuring VEGF and MMPs.

### Histology and morphometric analysis for assessing lung damage

Guinea pig lungs were fixed in 10% formalin and embedded in paraffin. The deparaffinized sections were stained with haematoxylin and eosin as described before [6]. Digital images were captured with Olympus CAMEDIA digital camera, Model C-7070 wide zoom (magnification × 10). The individual area (A) and the perimeter (P, the contour length) of each alveolar-airspace were identified and measured using NIH image software, as done before [6]. Based on these measurements, a perimeter to area ratio (P/A) was calculated for each alveolar-airspace. The P/A value was transferred into surface density S/V, using the morphometric relationship S/V = π/4 × P/A [6]. Two images were analyzed per lung section. Altogether 8 images were analyzed in 4 lung sections from each group.

### Oxidative protein damage as evidenced by immunoblotting

Oxidation of lung proteins was evidenced by immunoblotting of the dinitrophenylhydrazone derivatives of protein carbonyls followed by densitometric evaluation of the blots as described before [6], with the exception that whole lung lysates were used instead of microsomal membranes.

### Terminal deoxynucleotidyltransferase-mediated dUTP nick end labeling assay (TUNEL) assay of lung sections

The paraffin embedded tissue sections (5 μm) were deparaffinized, washed and permeabilized as mentioned above under histology and morphometric analysis. The TUNEL reaction was carried out using "In situ cell death detection kit, fluorescein" (Roche, USA) according to the manufacture's instruction, as described before [6]. The nuclei were counted by counter staining with 4, 6-diamidino-2-phenylindole (DAPI) at excitation wavelength, 350 nm. Two fields per section of four independent sections in each group were evaluated.

### Preparation of aqueous extract of cigarette smoke

Smoke from an Indian commercial filter-tipped cigarette (74 mm, tar content 20 mg, nicotine content 1 mg/cigarette) was extracted with 1 ml of 50 mM potassium phosphate buffer, pH 7.4, filtered through 0.22 μm Millipore filter and the pH adjusted to 7.4, as described before [7]. The aqueous extract of CS (AECS), thus obtained, was used immediately without delay.

### Isolation of para-benzosemiquinone (p-BSQ)

Para-benzosemiquinone (p-BSQ) has been isolated from AECS by differential solvent extraction, thin layer chromatography (TLC) and HPLC as faint yellow needle-shaped crystals (melting point 162°C).

AECS, freshly prepared from 20 cigarettes in 20 ml of buffer, as described above, was extracted thrice with equal volume of methylene chloride. To avoid any conversion of p-BSQ to para-benzoquinone (p-BQ) by autoxidation or disproportionation reaction in the aqueous medium, the process of methylene chloride extraction was made as quick as possible. The methylene chloride layer was discarded and the aqueous layer quickly extracted twice with 10 ml of n-butanol. The butanol layer was lyophilised and the residue extracted with 1 ml of acetone. The acetone was evaporated off in a speed vac and the residue dissolved in 120 μl of methanol. The methanol solution was subjected to preparative TLC using toluene: ethyl acetate (80:20, v/v) as the developing solvent. The band corresponding to R_f _0.26 was extracted with acetone, centrifuged and the supernatant dried in a speed vac. The yield of p-BSQ was 420–450 μg (purity, 98.5%), which was about 10% of the p-BSQ content of AECS, calculated on the value obtained by HPLC analysis. For further purification, the residue was dissolved in 200 μl water and extracted with 200 μl n-butanol. The butanol layer was collected and evaporated off in a speed vac. p-BSQ was further purified by HPLC using a Shimadzu 10A VP instrument attached to a UV detector and a chromatopac C-R6A. The column used was a normal phase silica column (Merck, LiChrospher^® ^Si 60, 25 cm, 5 μm). The mobile solvent was methylene chloride: methanol (90:10, v/v) and the flow rate, 0.5 ml/min. p-BSQ was detected at 293 nm at retention time (t_r_), 8.808 min (see Additional file 2, Fig.1 A, b) and that in AECS, 8.813 min (see Additional file 2, Fig.1 A, a). HPLC was also carried out using a reverse phase Shimadzu Shim-pack CLC-ODS (M) column (25 cm, 5 μm), where the mobile solvent was water: methanol (95:5, v/v) and the flow rate, 0.8 ml/min. p-BSQ was detected at 288 nm at t_r_, 13.458 min (see Additional file 2, Fig. 1 A, d) and that in AECS, 13.467 min (see Additional file 2, Fig.1 A, c). Although the resolution obtained with the ODS-column was better than that obtained with the silica column, the ODS-column could not be routinely used for analysing the AECS, because the ODS-column underwent degeneration after a few runs. The specific activity of p-BSQ at different stages of purification was monitored by measuring the formation of nmoles of carbonyl produced per mg BSA after incubation with one mg dry weight of each fraction. p-BSQ thus isolated was used without delay. When stored in the solid state, the potency of p-BSQ to produce carbonyl formation in bovine serum albumin (BSA) was lost 25% after one day, 50% after two days and 80% after 6 days. Separate HPLC analysis for measurement of p-BQ revealed that the p-BSQ isolated from AECS did not contain any p-BQ.

Apparently, p-BSQ is present exclusively in the tar phase of CS. The content of p-BSQ in AECS, as measured by HPLC, varied with the tar content. The lower the tar content, the lower was the amount of p-BSQ. Analysis of p-BSQ contents of 12 different conventional filter brand cigarettes from various parts of the world, including India, USA, England, Russia and Japan, gave the following values per cigarette: low tar (10–13 mg), 110–130 μg p-BSQ and medium tar (16–20 mg), 170–230 μg p-BSQ (n = 4). The results presented in this manuscript were obtained with p-BSQ isolated from a filter-tipped Indian commercial cigarette (tar content 20 mg, nicotine content 1 mg) having p-BSQ content of 220 ± 10 μg. The yield of purified p-BSQ was approximately 10%.

### Characterization of para-benzosemiquinone (p-BSQ)

p-BSQ was characterized by various physico-chemical analyses, including UV, mass, NMR and ESR spectroscopy (see Additional files 1, 2). In ESR spectroscopy, the g-value, calculated with reference to diphenyl picryl hydrazyl radical, has been found to be 2.004680, which is practically identical with the g-value of p-BSQ (2.004679), as reported before [19].

### Estimation of p-BSQ

p-BSQ isolated from AECS was quantitatively estimated either by UV spectrometry in aqueous solution (ε_288 _= 6.976 × 10^6 ^M^-1 ^Cm^-1^) or by HPLC analysis at 293 nm using a normal phase silica column (see Additional files 1, 2) The mobile solvent was methylene chloride: methanol (90:10, v/v), flow rate 0.5 ml/min, retention time 8.08 min, as described above under 'Isolation and characterization of p-BSQ'.

### Intratracheal instillation of p-BSQ in guinea pigs

Male guinea pigs weighing 800–900 g were used. All animal treatment procedures met the Institutional Animal Ethics Committee guidelines. One day before surgery, animals were treated with penicillin G (5000 units per day) and continued for five days for recovery of surgical stress. Before surgery, animals were starved for 24 h but given water *ad libitum*. Each animal was anesthetized by i.m. injection of ketamine hydrochloride (35 mg per kg body weight). A midline incision was made in the trachea and one end of a tube (Tygon tube. 020 ID. 060 OD 10, Small Parts, Inc. USA) was inserted into the trachea and the other end drawn beneath the skin to open up at the back of the animal's neck. After surgery, the guinea pigs were fed vitamin C supplemented diet (5 mg/day) for 7 days for recovery. After recovery, the guinea pigs were given vitamin C-free diet for 7 days followed by supplementation of 1 mg vitamin C/animal/day, as described before in the text. Following that, on the 8^th ^day a solution of 150 μg of p-BSQ in 200 μl normal saline was introduced in the trachea through the open end of the tube, twice daily, for 7 days. Sham controls received only saline (200 μl). Thereafter, the guinea pigs were sacrificed and the lung excised and analyzed.

### Measurement of p-benzoquinone (p-BQ)

p-BQ was measured by HPLC using a LichroCART 350-4, RP-18 (5 μm) column (Merck). p-BQ was detected at 245 nm at the retention time of 4.75 min using a mobile solvent of methanol: water (90:10, v/v) at a flow rate of 0.5 ml/min. The limit of detection was 500 pg.

### Measurement of vitamin C

Freshly excised lung tissue (0.5 g) was homogenized with 5% metaphosphoric acid (4.5 ml), centrifuged at 10,000 g for 10 min, the supernatant filtered through 0.22 μm Millipore filter and vitamin C was measured in the filtrate by HPLC after proper dilution with the mobile solvent. The column used was Lichro CART 250-4 NH_2 _(Merck); the mobile solvent, acetonitrile: 50 mM potassium dihydrogen phosphate solution (75:25, v/v); flow rate, 1.0 ml/min. Ascorbic acid was detected at 254 nm. The retention time of vitamin C was 6.1 min; limit of detection 500 pg.

### SDS-PAGE

Unless otherwise mentioned, guinea pig lung microsomal suspension equivalent to 1 mg protein was incubated with or without 50 μl of AECS or its equivalent amount of p-BSQ (90 nmoles) or p-BQ (45 nmoles) in 150 μl of 50 mM potassium phosphate buffer, pH 7.4, for 2 h at 37°C in air. SDS-PAGE was carried out as described before [7].

### Cell culture

A549 cells were grown to 50–60% confluence in HamF12 medium containing 10% fetal calf serum ((GIBCO-BRL, USA), 100 units/ml penicillin, 100 μg/ml streptomycin and 4 mM/ml glutamine.

### Terminal deoxynucleotidyl transferase-mediated deoxyuridine triphosphate nick end labelling (TUNEL) assay in A549 cells

A549 cells (2 × 10^6^) were treated with 50 μl of AECS, 180 nmoles of p-BSQ or 90 nmoles of p-BQ in culture medium at 37°C for 24 hours. The cells were then fixed with 4% p-formaldehyde and permeabilized with titron X-100 (0.1%) in 0.1% Na-citrate. The cells were then washed with PBS and subjected to the TUNEL assay using *in situ *cell death detection kit (Roche, USA), according to the manufacturer's instruction. The stained cells were counted under fluorescence microscope (Olympus B). Nuclei were simultaneously counted by counterstaining with 4', 6-diamidino-2-phenylindole dihydrochloride (DAPI, Sigma).

### Immunoblot analysis

Immunoblot analysis of the DNP-derivative of proteins was carried out as described before [6]. For analysis of VEGFR2 and MMP-9/MMP-12, 0.2 g lung tissue was homogenized in 1.8 ml lysis buffer (20 mM Tris {tris (hydroxymethyl) aminomethane chloride}), pH 7.4; 250 mM NaCl; 2 mM EDTA [ethylenediaminetetraacetic acid], pH 8.0; 0.1% Triton-X100; 0.01 mg/ml aprotinin; 0.005 mg/ml leupeptin; 0.4 mM phenylmethanesulfonyl fluoride [PMSF]; and 4 mM NaVO_4_. The homogenate was centrifuged at 19,064 g for 10 minutes and the supernatant (30 μg protein equivqlent) subjected to sodium dodecyl sulfate-polyacrylamide gel electrophoresis (SDS-PAGE). In the case of A549 cells, whole-cell extracts were prepared by lysing the AECS/p-BSQ/p-BQ-treated cells in lysis buffer. Lysates were then spun at 19,064 g for 10 minutes to remove insoluble material. Thirty to 50 μg of cytoplasmic protein extracts were resolved on 6–12% gel, as needed. After electrophoresis, the proteins were electro-transferred to a PVDF membranes, blocked with 5% nonfat milk (Bio-Rad), and probed separately with antibodies against VEGFR2, caspase3, Bax, Bcl-2 and PARP (1:1000) for 1 hour. Western blots of VEGF and MMP-12 were carried out with BALF proteins using antibodies of VEGF and MMP-12. Thereafter, the blots were washed, exposed to HRP-conjugated secondary antibodies for 1 hour, and finally detected by chemiluminescence.

### Cytochrome c detection

Five hundred mg lung tissue was homogenized in 4.5 ml of Tris buffer, pH 7.4 and centrifuged at 1000 g for 10 min at 4°C to sediment the nuclear fraction. The composition of the Tris buffer was 50 mM Tris-Hcl, 250 mM sucrose, 10 mM KCl, 2 mM EDTA, 1 mM PMSF, 1 mM DTT and protease inhibitor cocktail (Sigma, 1×). The supernatant was centrifuged at 10,000 g for 15 min at 4°C to recover the mitochondria. The resulting supernatant was kept as the cytosolic fraction. The mitochondria were washed twice with Tris buffer by centrifuging at 10, 000 g for 15 min and then resuspended in 400 μl of the same buffer. Protein was estimated in both the cytosol and the mitochondria to equalize samples before cytotochrome c detection by western blot. In western blot, 300 μg aliquots of mitochondrial and cytosolic proteins were separated on 12% SDS-PAGE gels, transferred to PVDF membrane and detected using HRP western blot detection system of Cell Signaling Technology.

### Immunofluorescence study for caspase 3 and Bax localization by Confocal microscopy

The paraffin embedded tissue sections (5 μm) were deparaffinized, washed and permeabilized as mentioned above under histology and morphometric analysis. Slides were blocked with 5% normal goat serum for 1 h and then incubated with rabbit polyclonal antihuman Bax antibody or anti cleaved caspase 3 antibody. After overnight incubation, the slides were washed and then incubated with goat antirabbit IgG for 1 h and counterstained for nuclei with DAPI for 5 min. Stained slides were mounted with mounting medium (Sigma Chemical) and analyzed under a confocal microscope (Zeiss LSM 510 META).

### Statistical Analysis

All values are expressed as mean ± SD. Statistical significance was carried out using a two factor ANOVA, with factors being CS and vitamin C, or one way ANOVA as needed. The P values were calculated using appropriate F-tests. Difference with P-values < 0.05 were considered significant.

## Results

### Cigarette smoke causes progressive lung damage: prevention by moderately large dosage of vitamin C

**Figure 1:**
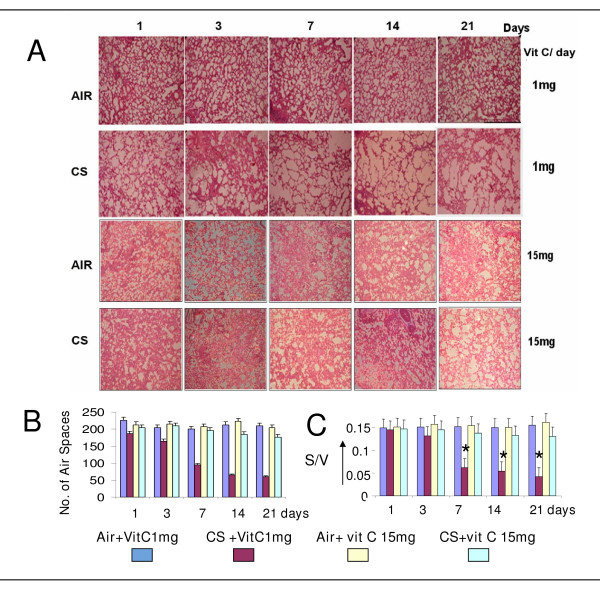
**Histopathology of lung sections of guinea pigs exposed to CS showing progressive lung damage and prevention by moderately large dosage of vitamin C.** A, Histology of lung sections of guinea pigs fed 1 mg or 15 mg vitamin C; Air, sham controls exposed to air; CS, exposed to CS; stained by hematoxylin and eosin. The number of guinea pigs used in each group was 4. Eight images were analyzed in 4 lung sections (2 images/section/animal) from each group (magnification ×10). B, C, Morphometric Measurements of the number of alveolar air space and surface density (S/V). Values are ± S.D. S/V has been calculated by the formula S/V (surface density) = π/4 × P/A, where P is the mean perimeter (contour length) of air space and A is the area of air space [6]. The number of air spaces and the S/V values calculated after 7, 14 and 21 days of smoke exposure are significantly less (p < 0.05)than that observed after 1 day exposure; * over the bars (Fig. 1 C) indicate p < 0.05 with respect to controls.

Histology (Figure 1A) shows that CS-exposure causes progressive damage of vitamin C-restricted (CS + vit C 1 mg) guinea pig lungs on and from the 7th day of smoke exposure. The severity of the lesion increases up to 21^st ^day of the experimental period (Fig 1). Lung damage has been evidenced by enlargement of alveolar air spaces accompanied by morphometric changes. Figs 1B, C show morphometric measurements of the alveolar air space calculated from 8 different images from each group, including the mean air space and the surface density (S/V, surface per unit volume) per image. The surface density has been calculated using the formula: S/V = π/4 × P/A, where P is the perimeter (contour length) of air space and A is the total area of the air space [6]. It is known that exchange of oxygen and carbon dioxide of alveolar cells is largely regulated by surface density [6]. Fig. 1C shows that compared to the S/V value (0.150 ± 0.017 SD) of air-exposed sham controls (Air + vit C 1 mg), there is progressive and significant decrease (p < 0.05) in the S/V values of the CS-exposed guinea pigs fed 1 mg vitamin C/day (CS + vit C 1 mg). The values are 0.065 ± 0.021 after 7 days; 0.056 ± 0.020 after 14 days and 0.042 ± 0.020 after 21 days. However, when instead of 1 mg vitamin C, the guinea pigs were fed a moderately large dose of the vitamin (15 mg/day) and subjected to CS exposure (CS + vit C 15 mg), there was no significant lung damage as evidenced by the prevention of morphometric change and no inhibition in the S/V values (Fig. 1B, C). The results indicate that supplementation of a moderately large dose of vitamin C prevents CS-induced lung damage. A dose of 5 mg vitamin C/day was incapable to prevent the lung damage (data not shown). In the sham controls exposed to air, the dose of 15 mg vitamin C (Air + vit C 15 mg) did not practically affect the morphology of the alveolar cells and the S/V ratio (Fig. 1A, B, C).

Lung damage caused by CS exposure to guinea pigs for 21 days was apparently irreversible. Histology revealed that the damage was not repaired after discontinuation of the smoke for a period of 4 weeks (data not shown). Also, once the lung was damaged by exposure to CS, administration of 15 mg vitamin C/day could not reverse the process.

That exposure of guinea pigs fed 1 mg vitamin C/day to cigarette smoke caused lung destruction was evidenced by increase in mean linear intercept (Lm) and destructive index (DI) of the lung sections. In separate experiments, both right and left lungs (middle lobes) of three guinea pigs exposed to smoke for 14 days were fixed by intra-tracheal inflation with 10% buffered formalin at a constant pressure of 20 cm water for 24 hours. After routine processing of successive dehydration, the lungs were embedded in paraffin. Six five μm sections from each lobe were stained with hematoxylin-eosin. Four representative nonoverlapping images from each section were captured in a Olympus BX40 microscope at 200 magnification. Lm was measured by the method of Robbesom et al. [20] and DI was measured by the point counting method of Saetta et al. [21]. There was significant destruction of the lungs after exposure to CS for 14 days. Lm and DI of CS-exposed animals were 0.3511 ± 0.0415 (Lm) and 60.1 ± 7.1 (DI) and that of sham controls 0.3041 ± 0.0174 (Lm) and 33.4 ± 12.9 (DI), respectively. P values (CS vs control) for both Lm and DI are < 0.05.

It is known that cigarette smoke causes local inflammation in the lung, which is reflected by an increase in the number of neutrophils in the bronchoalveolar lavage fluid (BALF) [22]. Here we have observed that in the BALF of guinea pigs (n = 3) exposed to CS for 14 days, both the total number of leucocytes (225 ± 30 SD × 10^4^) and the neutrophils (135 ± 20 × 10^4^) increased significantly (p < 0.05) over that of sham controls (leucocytes 160 ± 10 × 10^4^; neutrophils 23 ± 3 × 10^4^).

### Vitamin C status in the lungs of CS-exposed guinea pigs

After 7 days of vitamin C deficiency, the vitamin C content of the guinea pig lung was 0.24 mM ± 0.02 SD (n = 4). When these guinea pigs were fed 15 mg vitamin C/day, the vitamin C content increased to 1.45 mM ± 0.13 SD and the value did not decrease significantly after exposure to CS for 7 days (Table 1). Similar observations were made after exposure to CS for 14 and 21 days, respectively (Table 1). On the other hand, when the guinea pigs were fed 1 mg vitamin C/day, the vitamin C content was 0.31 mM ± 0.02 SD, which after CS-exposure fell to 0.10 mM ± 0.01 SD (Table 1). CS consumes vitamin C [7], so the fall in the vitamin C level was apparently due to consumption of vitamin C by CS. This vitamin C level was inadequate for preventing CS-induced lung damage. A dose of 5 mg vitamin C/day (lung content 0.85 mM ± 0.08 SD) was also inadequate, because after exposure to CS, the value decreased to 0.30 mM ± 0.04 SD (Table 1). This would explain why a moderately large dose of vitamin C (15 mg/day) is needed to protect the guinea pigs from CS-exposed lung damage. This supports our earlier observation that in guinea pigs a dosage of 15 mg vitamin C/day almost completely prevents CS-induced protein oxidation in the lungs *in vivo*, but 5 mg vitamin C/day fails to do so [8].

**Table 1:** Vitamin C levels in the lung tissues of vitamin C-restricted (1 mg/day) and vitamin C-supplemented (5 mg/day & 15 mg/day) guinea pigs before and after CS exposure

**Days of CS exposure**	**Vitamin C supplemented Groups**					
	
	**1 mg supplemented group**		**5 mg supplemented group**		**15 mg supplemented group**	
	**Vitamin C level (mM)**		**Vitamin C level (mM)**		**Vitamin C level (mM)**	

	**Before CS exposure**	**After CS exposure**	**Before CS exposure**	**After CS exposure**	**Before CS exposure**	**After CS exposure**

**7**	0.31 ± 0.02*	0.10 ± 0.01	0.85 ± 0.08	0.30 ± 0.04	1.45 ± 0.13	1.31 ± 0.14

**14**	0.22 ± 0.02	0.10 ± 0.01	0.80 ± 0.08	0.28 ± 0.03	1.42 ± 0.13	1.30 ± 0.12

**21**	0.20 ± 0.02	0.10 ± 0.01	0.80 ± 0.08	0.28 ± 0.03	1.40 ± 0.13	1.28 ± 0.12

Dietary supplementation of vitamin C was initiated after deprivation of the vitamin for 7 days. After 7 days of deprivation, the vitamin C content of the lung was estimated to be 0.24 ± 0.02 mM SD (n = 4).

* Data represent ± S.D (n = 4). Vitamin C was measured by HPLC as described in Methods.

### CS- induced apoptosis in the guinea pig lung *in vivo*

Apoptosis has been evidenced by terminal deoxynucleotidyltransferase-mediated dUTP nick end labeling (TUNEL) assay, activation of caspase 3, cleavage of poly ADP ribose polymerase (PARP) and increase in the Bax/Bcl-2 ratio of the guinea lung tissue.

### Terminal deoxynucleotidyltransferase-mediated dUTP nick end labeling (TUNEL) assay

**Figure 2:**
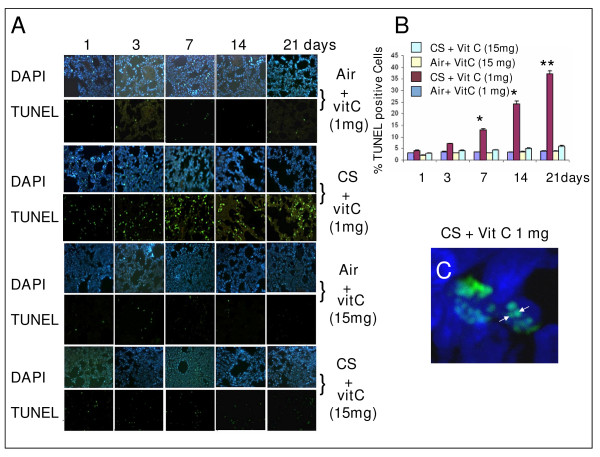
**A, Detection of DNA strand breaks by TUNEL assay in lung cells of vitamin C-restricted (1 mg vitamin C/animal/day) or vitamin C-supplemented (15 mg/animal/day) guinea pigs exposed to air CS (as described under Methods).** Following CS exposure, the guinea pigs were sacrificed after 1, 3, 7, 14 and 21 days. Lower Panel: the lung sections were stained with fluorescein labeled dUTP according to the protocols discussed under Methods. Green fluorescence indicates TUNEL positive cells. Upper Panel: Lung sections corresponding to the upper panel were stained with DAPI to identify the cell nuclei. B, Quantitative evaluation of TUNEL positive cells in lungs of guinea pigs exposed to air or CS. Eight images were analyzed in 4 lung sections (2 images/section/animal) from each group, respectively; the bars over the respective columns represent means ± SD; * indicates p < 0.05 and ** indicates p < 0.001 with respect to sham controls. C, Colocalization of TUNEL and DAPI staining in the nuclei of lungs of guinea pig fed 1 mg vitamin C/day and exposed to CS for 21 days; original magnification × 63; image captured by confocal microscopy (Zeiss LSM 510 META).

Fig. 2A shows that compared to air-exposed animals fed 1 mg vitamin C per day (Air + vit C 1 mg), there is progressive increase in the TUNEL positive cells in the lungs of guinea pigs fed 1 mg vitamin C/day and exposed to CS (CS + vit C 1 mg) for a period up to 21 days. The upper panel of each group shows the nuclei stained with DAPI. The TUNEL positive cells are marked by green fluorescence attributable to fluorescein-dUTP labelling in the lower panel of each group, namely, Air + vit C 1 mg (sham controls, air exposed and fed 1 mg vitamin C/day), Air + vit C 15 mg (air exposed and fed 15 mg vitamin C/day), CS + vit C 1 mg (CS exposed and fed 1 mg vitamin C/day) and CS + vit C 15 mg (CS exposed and fed 15 mgvitamin C/day), respectively. Quantitative evaluation of the extent of DNA fragmentation (Fig. 2B) indicates that compared to the air-exposed animals, there is a gradual increase in the TUNEL positive cells in the lungs of guinea pigs fed 1 mg vitamin C/day and exposed to CS up to 21 days. The number animals used in each group were 4. Images from 8 different lung sections were examined from each group. The per cent of TUNEL positive cells in lung sections of guinea pigs exposed to air and fed 1 mg vitamin C/day (Air + vit C 1 mg) for 7, 14 and 21 days are 3, 4 and 5, respectively (Fig. 2B). In contrast to this, the per cent of TUNEL positive cells in the CS-exposed animals fed 1 mg vitamin C/day (CS + vit C 1 mg) are significantly increased (p < 0.05 after 7 and 14 days and p < 0.001 after 21 days of CS exposure). The values are 14 ± 1 (mean ± SD) after 7 days, 25 ± 2 after 14 days and 38 ± 3 after 21 days (Fig. 2B). However, when the CS exposed guinea pigs are fed 15 mg vitamin C/day (CS + vit C 15 mg), there is practically no increase in the TUNEL positive cells (Fig. 2A, B). Also, the per cent of TUNEL positive cell is negligible in air exposed animals fed 15 mg vitamin C/day (Fig. 2B). Fig. 2C depicts DNA fragmentation by confocal microscopy at the single cell level in nuclei of lung cells of guinea pigs fed 1 mg vitamin C and exposed to CS for 21 days. No such DNA fragmentation was observed in the lung cells of CS-exposed guinea pigs fed 15 mg vitamin C/day (Fig. not shown).

### Activation of caspase 3

**Figure 3:**
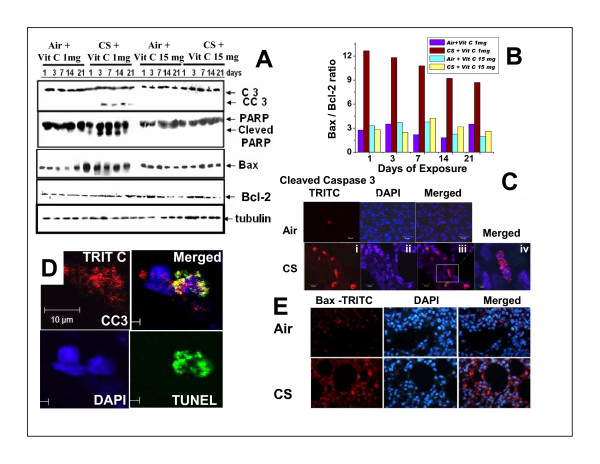
**A, Immunoblots of caspase 3, PARP, Bax and Bcl-2 of the lung extracts of guinea pigs exposed to air or CS supplemented with 1 mg or 15 mg vitamin C/animal/day.** Vit C means vitamin C. Air + vit C means exposed to air and fed 1 mg or 15 mg vitamin C/day; CS + vit C, means exposed to CS and fed 1 mg or 15 mg vitamin C/day. B, quantitative evaluation of Bax/Bcl-2 ratio. C, detection of apoptosis in the lung of guinea pig fed 1 mg vitamin C and exposed to CS for 21 days by immunofluorescence analysis using cleaved caspase 3 antibodies; lower panel I, stained with TRITC (red signal); II, stained with DAPI (blue); III, double staining with DAPI and TRITC; IV, enlarged version of III. D, colocalization of cleaved caspase 3 (CC3) in lungs of CS-exposed guinea pigs fed 1 mg vitamin C; red signal (TRITC) indicates cleaved caspase 3; blue signal, DAPI and green signal, TUNEL. Merged mens combination of TRITC, DAPI and TUNEL; image captured by laser scanning confocal microscope; original magnification × 63. E, Accumulation of Bax proteins in the lungs of CS-exposed guinea pigs fed 1 mg vitamin C/day; red signal, stained with TRITC and blue signal, stained with DAPI. Merged means combination of TRITC and DAPI.

The CS-induced apoptosis has been further confirmed by the activation of caspase 3 in the guinea pig lung tissue. Fig. 3A shows that the levels of cleaved products of caspase 3 (CC3, 17 KDa) are increased in CS-exposed guinea pigs supplemented with 1 mg vitamin C/day. The increase is appreciable after 3, 7 and 14 days of CS exposure. After 21 days of CS exposure, the increase is somewhat less but detectable. The level of active caspase 3 is practically undetectable in pair fed air- exposed sham control as well as in guinea pigs fed 15 mg vitamin C/animal/day (Fig. 3A). The immunoblot analysis of active caspase 3 is further supported by immunofluorescence study using cleaved caspase 3 specific antibodies (Fig. 3C). Red signals (inset Fig 3I) indicate cleaved caspase 3 after staining with tetramethyl rhodamine iso-thiocyanate (TRITC). Blue signals represent DAPI-stained nuclei. The inset (Fig. 3C III) depicting the red signals represent cleaved caspase 3 at the level of a single cell, an enlarged version of which is shown in Fig. 3C IV. The presence of cleaved caspase 3 (CC3) in the nuclei of CS-exposed lung cells is further confirmed by confocal microscopy (Fig. 3D), where staining is depicted separately by TRITC, DAPI, TUNEL as well as merging of all the three stains.

### Cleavage of poly ADP ribose polymerase (PARP)

The CS-induced apoptosis has also been evidenced by proteolytic cleavage of PARP, a substrate for activated caspase 3. Fig. 3A shows that the levels of cleaved products of PARP are markedly increased in the lungs of CS-exposed guinea pigs fed 1 mg vitamin C/animal/day. Cleaved PARP is undetectable in pair fed air-exposed sham control as well as in CS-exposed guinea pigs fed 15 mg vitamin C/animal/day.

### Bax/Bcl-2 ratio

Fig 3A shows that the levels of Bax protein are increased markedly in the lungs of guinea pigs fed 1 mg vitamin C/animal/day and exposed to CS. Fig 3A further shows that in contrast to Bax, the levels of Bcl-2 are not increased in the lungs of guinea pigs fed 1 mg vitamin C/day and exposed to CS. This resulted in an increase of the Bax/Bcl-2 ratio. Using densitometric scanning by Image Quant TL software, the Bax/Bcl-2 band abundance ratios in the CS + vit C 1 mg group have been determined to be 12.66 after 1 day, 11.81 after 3 days, 10.78 after 7 days, 9.23 after 14 days and 8.70 after 21 days of CS exposure (Fig. 3B). The figure (Fig. 3B) also shows that 15 mg vitamin C completely prevents the increase of Bax protein in CS-exposed guinea pigs (CS + vit C 15 mg group) resulting in no increase of the Bax/Bcl2 ratio. The ratio of Bax/Bcl-2 in the CS + vit C 15 mg group has practically no difference with either Air + vit C 1 mg group or Air + vit C 15 mg group (Fig 3B). The up regulation of Bax protein in CS-exposed guinea pig lung has also been documented by immunoflouresence study (Fig. 3E). The red signal stained with TRITC corresponds to Bax protein.

### Protein oxidation is an early event of CS exposure

**Figure 4:**
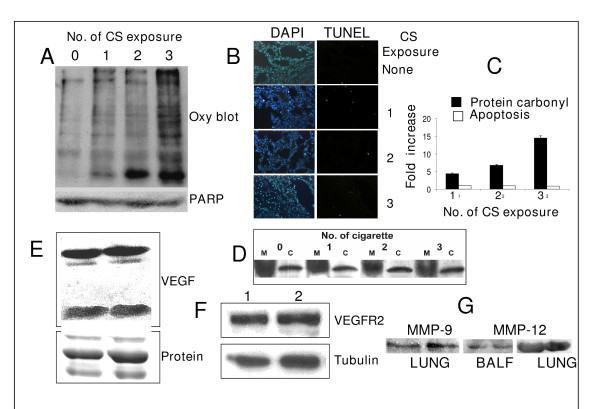
**A, Upper panel: Immunoblots of the DNP-derivatives of lung proteins of guinea pigs exposed to smoke from 1, 2 and 3 cigarettes.** Twenty five μg proteins isolated from air-exposed or CS-exposed guinea pigs fed vitamin C-free diet for 7 days were converted to the DNP-derivative followed by immunoblotting as mentioned in the Methods. Lower panel: The membrane was reprobed with anti PARP antibodies which show that there was no PARP cleavage. B, Detection of DNA strand breaks (TUNEL assay) in lung cells of guinea pigs exposed to smoke from 1, 2 and 3 cigarettes. C, Quantitative evaluation of TUNEL assay and protein carbonyl formation in the lungs of guinea pigs exposed to smoke from 1, 2 and 3 cigarettes. Protein carbonyl was measured by densitometric scanning using Quantity One- 4.4 (Bio-Rad) Software. Bars over the respective columns represent means ± SD (n = 4). D, Western blots of mitochondrial and cytosolic cytochrome c of lungs of guinea pigs exposed to 1, 2 and 3 cigarettes; M represents mitochondria and C, cytosol. E, Immunoblots of VEGF in BALF isolated from guinea pigs with and without exposure to smoke from 3 cigaretes. Lane 1, sham control after exposure to air; lane 2, after exposure to 3 cigarettes; Similar results were obtained from 3 different animals. The upper panel represents the dimmer of VEGF (Mr 42 kDa) and lower panel, the monomer (Mr 21 kDa). Loading control (SDS-PAGE) is shown at the bottom. F, Immunoblots of VEGRF2 in lung tissue of guinea pigs with and without exposure to smoke from 3 cigarettes. Lane 1, sham control after exposure to air; lane 2, after exposure to 3 cigarettes; lower panel indicates loading control. Similar results were obtained from 3 different animals. G, western blots showing levels of MMP-9 and MMP-12 in lung tissue and BALF isolated from guinea pigs fed 1 mg vitamin C/day and exposed to air or CS for 14 days; In each of the blots under MMP-9 and MMP-12, the left blot indicates exposed to air and the right, exposed to CS.

Earlier we had shown that when guinea pigs fed 1 mg vitamin C/day were exposed to CS, there was progressive formation of protein carbonyl on and from the first day of CS exposure at an exposure rate of 5 cigarettes/guinea pigs/day [6]. Here we show that protein carbonyl formation starts even after exposure of the guinea pigs to smoke from only one cigarette (Fig. 4A, lane 1). The extent of carbonyl formation increased markedly after exposure to smoke from 2 and 3 cigarettes (Fig. 4A, lane 3). Bottom portions of the oxyblots of lanes 1, 2 and 3 in Fig. 4A show protein carbonyls of degraded proteins. Fig. 4C shows quantitative increase in protein carbonyl formation as a function of smoke exposure, as measured by densitometry scanning. However, in contrast to marked increase in the protein carbonyl formation, no apoptosis was observed in the lungs of guinea pigs exposed to smoke from 3 cigarettes, as evidenced by lack of increase in the number of TUNEL positive cells (Fig. 4B, C) and cleavage of PARP (Fig. 4A, lower panel). These results confirm our previous hypothesis that protein modification precedes apoptosis [6].

A growing body of evidence supports that oxidative stress activates or induces expression of the pro-apoptotic Bcl-2 family member Bax, which in turn triggers the release of cytochrome c from mitochondria into the cytosol where it binds to APAF-1 and participates in the activation of caspases that leads to apoptosis (23–30). We have observed that while Bax is activated in the lungs of guinea pigs after exposure to CS from 5 cigarettes (1 day of exposure, Fig. 3A), no Bax activation is noticed after exposure to smoke from 1, 2, or 3 cigarettes (data not shown). Also, western blot analysis demonstrates that there is no decrease of mitochondrial cytochrome c and increase in cytosolic cytochrome c in the lungs of guinea pigs exposed to smoke from 1, 2 or 3 cigarettes and (Fig. 4D). These results together with the observation that protein oxidation takes place after exposure to smoke from 1 cigarette indicate that protein oxidation precedes Bax activation and translocation of cytochrome c.

It is also reported that vascular endothelial growth factor (VEGF) and VEGF receptor (VEGFR2) expressions decrease significantly in rodent models after prolonged exposure to CS [31,32]. In other studies, emphysema was induced by inactivating VEGF and VEGFR2 [33,34]. Here we show that decreases in the expressions of VEGF and VEGFR2 at the protein levels are not the initial events. While the level of protein carbonyl increased markedly in the lung after exposure of the guinea pigs to smoke from 3 cigarettes (Fig. 4A, C), there was no decrease in the level of VEGF in the broncho-alveolar lavage fluid (BALF) or VEGFR2 in the lung tissue, as evidenced by western blot (Fig. 4E, F).

Matrix metalloproteases (MMPs) are believed to be important in the pathogenesis of cigarette smoke-induced emphysema. It has been shown that an MMP-9/MMP-12 inhibitor can substantially ameliorate morphological emphysema in guinea pigs, suggesting that MMPs may be important mediators of the anatomical changes behind COPD [35]. Here we demonstrate that MMP-9 is markedly elevated in the lungs of guinea pigs fed 1 mg vitamin C/day and exposed to CS for 14 days (Fig 4G). Densitometric scanning using Image Quant TL software show that the band abundance in CS-exposed guinea pigs is 1562 (arbitrary unit) compared to 979 in sham controls. No MMP-9 has been detected in the BALF. Fig. 4G further shows that there is no appreciable change in the levels MMP-12 either in the BALF or in the lungs of guinea pigs after exposure to CS.

### N-acetylcysteine (NAC) is partially effective in preventing CS-induced protein modification, apoptosis and lung damage

**Figure 5:**
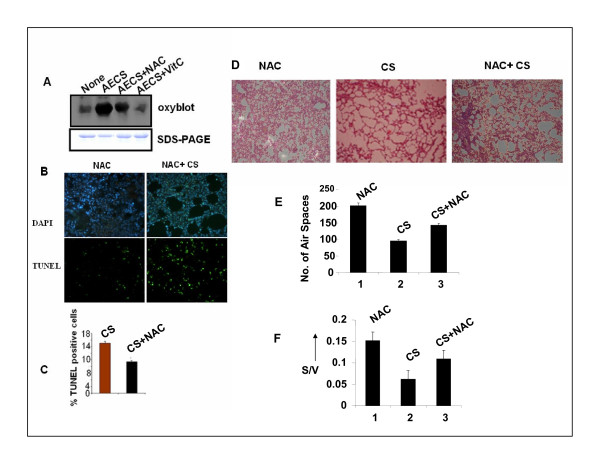
**Effect of N-acetyl cysteine (NAC) on CS-induced protein oxidation *in vitro *and apoptosis and lung damage in guinea pigs *in vivo*.** A, CS-induced protein carbonyl formation in BSA in the presence and absence of NAC and vitamin C, as measured by immunoblot analysis. The incubation mixture contained BSA (1 mg) AECS (50 μl), NAC (100 μM) or vitamin C (200 μM) in a final volume of 200 μl of 50 mM potassium phosphate buffer, pH 7.4; incubated for 2 h at 37°C with shaking. After incubation, production of the DNP derivative was measured by immunoblot analysis, as describe before [6]. B, Detection of DNA strand breaks in lung cells of guinea pigs exposed to air or CS in the presence of NAC by TUNEL assay. After feeding vitamin C-free diet for 7 days, the guinea pigs were supplemented with 1 mg vitamin C/day and fed 15 mg NAC/animal/day and exposed to air or CS for 7 days, as described under Methods. C, Quantitative evaluation of TUNEL positive cells in lungs of guinea pigs exposed to CS in the presence or absence of NAC. Eight images were analyzed in 4 lung sections (2 images/section/animal) from each group, respectively; bars over the respective columns represent means ± SD. D, Histopathology profiles of lung sections of guinea pig after exposure to CS in the presence and absence of NAC (15 mg/animal/day). The number of guinea pigs used in each group was 4. Eight images were analyzed in 4 lung sections (2 images/section/animal) from each group (magnification ×10). E, F, Morphometric Measurements of alveolar air space, and surface density (S/V). Values are ± SD; number of images analyzed in each group:8.

N-acetylcysteine (NAC), a known antioxidant, has been used in the treatment of emphysema with controversial results [14-16]. Here we show that at similar concentrations, NAC is less effective than vitamin C in preventing CS-induced protein oxidation, apoptosis and lung damage (Fig. 5). Fig. 5A shows that while vitamin C completely prevents AECS-induced oxidation of bovine serum albumin (BSA) *in vitro*, NAC is only partially effective. Densitometric analysis of the western blot (Fig. 5A) revealed that NAC prevented CS-induced BSA oxidation only about 40%. Fig 5B, C show that as observed in the case of oxidative protein damage (Fig 5A), CS-induced apoptosis is also partially prevented by NAC. Quantitative evaluation of the TUNEL positive cells in the lungs of guinea pigs exposed to CS for 7 days indicate that NAC prevents DNA fragmentation only about 37% (Fig 5B, C). Also, in contrast to vitamin C, NAC is partially effective in preventing the lung damage. Morphometric measurements of the alveolar air space and surface density (S/V) indicate that CS exposure to guinea pigs fed 1 mg vitamin C for 7 days produced about 58% lung damage. When the guinea pigs were fed NAC at a dose of 15 mg/animal/day along with CS exposure, the lung damage was prevented to the extent of only 33% (Figs. 5D, E, F). Although this prevention (33%) is significant (p < 0.05), it is much less than that obtained with vitamin C, which almost completely prevents CS-induced lung damage.

### p-Benzosemiquinone (p-BSQ) mimics AECS for oxidative protein modification and proteolysis *in vitro*: prevention by vitamin C

**Figure 6:**
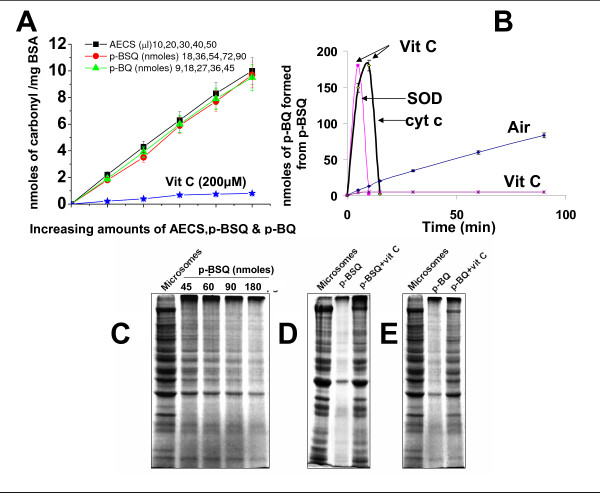
**p-BSQ mimics AECS causing oxidative modification and proteolysis in vitro: prevention by vitamin C.** A, Dose-dependent AECS/p-BSQ/p-BQ-induced protein carbonyl formation in BSA in the presence or absence of vitamin C, as measured spectrophotometrically. The incubation mixture was same as in Fig.5A using AECS (50 μl), p-BSQ (90 nmoles) or p-BQ (45 nmoles). Small bars over the points in the graph represent mean ± SD. B, Conversion of p-BSQ to p-BQ by disproportionation reaction or by SOD/cytochrome c catalysed oxidation. p-BSQ (180 nmols) was incubated in 200 μl of 50 mM phosphate buffer, pH 7.4 at 37°C in air with or without the presence of Cu, Zn-SOD (30 μg) and cytochromec (180 nmoles), incubation was carried out in air. Aliquots were taken, diluted with mobile solvent and p-BQ estimated by HPLC. Details are given in Methods. ↓ Indicates addition of vitamin C (200 μM). C, Degradation of guinea pig lung microsomal proteins as a function of p-BSQ concentration. D, E, Degradation of guinea pig lung microsomal proteins by p-BSQ (180 nmoles) and p-BQ (90 nmoles) in the presence and absence of vitamin C (200 μM). Conditions are same as described previously [7]. Similar observations were made in three independent experiments.

Considering that p-BSQ, a long-lived radical of CS, may be a major cause of CS-induced protein oxidation, we have isolated, characterized and compared the effect of p-BSQ with that of AECS on protein carbonyl formation in BSA. Oxidized protein that reacts with 2, 4-dinitrophenyl hydrazine (DNPH) forming DNP-derivatives is widely used as a measure of carbonyl formation produced by oxidative modification [36,37]. HPLC analysis revealed that 50 μl of AECS contained approximately 90 nmoles of p-BSQ. Fig. 6A shows that the protein carbonyl formed (9.7 ± 0.8 nmoles, n = 3) by incubation of 1 mg BSA with 90 nmoles of p-BSQ is almost similar to that produced (10.0 ± 1.0 nmoles) by incubation of 1 mg BSA with 50 μl of AECS. This indicates that p-BSQ mimics AECS in inducing BSA oxidation. Tracing to the mechanism of p-BSQ-mediated protein oxidation we have observed that at pH 7.4, about one half of p-BSQ is converted to p-benzoquinone (p-BQ) (Fig. 6B). The rate of conversion of p-BSQ to p-BQ is very rapid in the presence of transition metal containing proteins such as Cu, Zn-SOD and cytochrome c (Fig. 6B). Dose-depended effects of AECS, p-BSQ and p-BQ on BSA oxidation *in vitro *show that similar protein oxidation takes place when AECS is replaced by its content of p-BSQ or p-BQ produced from p-BSQ (Fig 6A). The protein carbonyl formation by AECS, p-BSQ or p-BQ is inhibited by 200 μM vitamin C (Fig. 6A). This is apparently because vitamin C reduces p-BQ (Fig. 6B) and thereby prevents protein carbonyl formation.

Our consideration that major portion of the p-BSQ-induced protein carbonyl is produced via the formation of p-BQ is supported by the observation that p-BSQ forms quinone-Michael adduct with BSA. It is known that p-BQ forms Michael adduct with free amino groups of proteins at the room temperature (38, 39). We performed MALDI-TOF-MS analyses of BSA before and after incubation with p-BSQ or p-BQ at pH 7.4. We observed that incubation of 100 μg of BSA (MW 66,340 Da) with 185 nmoles of p-BQ produced an adduct of MW 68,347 Da, indicating association of 19 nmoles of p-BQ to in the BSA molecule (see Additional file 3, Fig. 2). When p-BQ was replaced by p-BSQ, the MW of the product was found to be 67,292 DA, indicating the addition of 9 nmoles of p-BQ. The increased number of p-BQ in the adduct obtained after incubation of p-BQ with BSA is apparently because p-BQ reacts directly with BSA, while p-BSQ produces adduct via formation of p-BQ. The BSA-Michael adducts produced from p-BSQ or p-BQ readily forms DNP-derivatives after reacting with DNPH. This shows that the BSA-Michael adduct contains carbonyl groups, which is a measure of oxidative protein damage. The formation of Michael adducts either from p-BSQ or p-BQ is completely prevented by 200 μM vitamin C (data not shown).

An intriguing aspect of protein modification is that modified proteins become vulnerable to degradation by proteases present in their vicinity [7]. Guinea pig lung microsomes contain proteases and we had previously shown that incubation of guinea pig lung microsomes with AECS causes extensive protein degradation [7]. Here we show that microsomal protein degradation is a function of the concentration of p-BSQ (Fig 6C). Similar protein degradation is observed when p-BSQ (180 nmoles) is replaced by p-BQ (90 nmoles) (Fig 6D, E). Vitamin C (200 μM) prevents p-BSQ/p-BQ-induced microsomal protein degradation apparently by reducing and thereby by inactivating p-BQ.

### p-BSQ induces apoptosis in A549 lung epithelial cells

**Figure 7:**
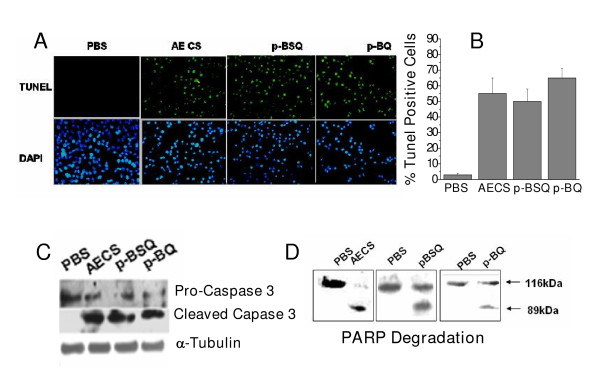
**AECS/p-BSQ/p-BQ-induced apoptosis in A549 lung epithelial cells *ex vivo*.** A, Detection of DNA strand break using TUNEL assay. The cells were examined using a fluorescence microscope (Olympus B, magnification × 20). Digital images were captured with cool CCD camera (Olympus). Upper panel, TUNEL positive cells; lower panel, nuclei stained with DAPI. About 10 fields (150 cells/field) of three independent experiments were evaluated. A549 cells (2 × 10^6^) were treated with 50 μl of AECS, 180 nmoles p-BSQ or 90 nmoles p-BQ in culture medium at 37°C for 24 h. B, Quantitative evaluation of TUNEL positive cells treated with AECS/p-BSQ/p-BQ; PBS indicates control treated with phosphate buffered saline; small bars over the columns indicate ± SD. C, Activation of pro-caspase-3 analyzed by immunoblotting. D, Cleavage of poly ADP ribose polymerase (PARP) analysed by immunobloting using anti PARP antibody. For immunoblot assay of caspase 3 and PARP, cells were treated with AECS, p-BSQ or p-BQ for 12 h. Similar observations were made in three independent experiments.

We have shown above in this paper that the initial event of CS exposure to guinea pigs is oxidative protein damage in the lungs, which is followed by apoptosis. We have also shown that p-BSQ mimics AECS in causing protein oxidation (Fig. 6A). Here we further demonstrate that p-BSQ mimics AECS in inducing apoptosis in A549 cells. Similar to that noticed in the lung cells of guinea pigs exposed to CS *in vivo*, apoptosis has been evidenced in A549 cells by DNA fragmentation (TUNEL assay) (Fig. 7A, B), activation of caspase 3 (Fig. 7C) and cleavage of poly (ADP-ribose) polymerase (PARP) (Fig. 7D). Since p-BSQ induces protein oxidation via the formation of p-BQ, we have also investigated the effect of p-BQ on apoptosis in A549 cells. Figs. 7A and 7B show that compared to PBS-treated cells, the per cent of cells with DNA damage is significantly high (p < 0.001, n = 3) following treatment of A549 cells with 50 μl of AECS (45% ± 7), 180 nmoles of p-BSQ (54% ± 7) or 90 nmoles of p-BQ (73% ± 7) for 24 hours. The lower panel of each group in Fig. 7A shows the nuclei stained with DAPI. That p-BSQ or p-BQ mimics AECS in causing apoptosis is further confirmed by the levels of cleaved products of caspase 3 (Fig. 7C) and PARP (Fig. 7D). AECS, p-BSQ or p-BQ- induced DNA fragmentation, activation of caspase 3 and cleavage of PARP were prevented by 200 μM vitamin C (data not shown).

### p-BSQ causes apoptosis and lung damage in guinea pigs *in vivo*: prevention by vitamin C

**Figure 8:**
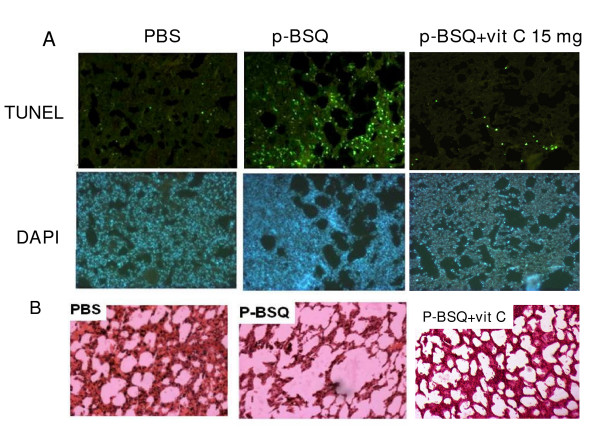
**Apoptosis and lung damage caused by intratracheal instillation of p-BSQ in guinea pigs and prevention by vitamin C.** The guinea pigs were given a solution of 150 μg p-BSQ in 200 μl of normal saline/guinea pig/twice a day for 7 days by intratracheal instillation and then sacrificed. Details of intratracheal instillation are given in Materials and Methods. Control animals (PBS treated) received only saline (200 μl). Supplementation of vitamin C (15 mg/day) prevented apoptosis (about 80%) and lung damage (about 85%) in the p-BSQ treated group. In the saline-treated sham controls, administration of vitamin C (15 mg/day) did not produce any significant change in the morphology of alveolar cells or TUNEL assay (Fig. not shown). A, Upper panel shows TUNEL positive cells; lower panel, lung sections stained with DAPI. The number of guinea pigs used in each group was three. Six images were analyzed in three lung sections (2 images/section) from each group (magnification × 10). B, Histopathology (H and E staining) of lung sections after intratracheal instillation of PBS or p-BSQ. Marked enlargement of alveolar air spaces is found in the p-BSQ group (magnification × 20), which is markedly inhibited by vitamin C.

We had shown before that exposure of guinea pigs to CS causes apoptosis, which is accompanied by lung damage within 7 days of smoke exposure [6]. Apoptosis is a hallmark of emphysema [2]. Here we demonstrate that in guinea pigs, intratracheal instillation of a solution of p-BSQ (150 μg/guinea pig/twice a day) in normal saline (200 μl) for 7 days results in apoptosis and lung damage. Apoptosis has been evidenced by TUNEL assay (Fig. 8A, upper panel). Lower panel depicts DAPI staining. Quantitative evaluation of apoptosis in lungs of guinea pigs fed 1 mg vitamin C per day shows that compared to saline treatment, the percentage of TUNEL positive cells in p-BSQ-treated animals is significantly high (p < 0.05). The percentage of DNA damaged cells after p-BSQ treatment is 26 ± 2 SD (n = 3) and that after saline treatment is 3 ± 1. Administration of 15 mg vitamin C inhibited apoptosis about 73% (Fig. 8A, upper panel). Lung damage has been manifested by morphometric change and enlargement of air spaces (Fig. 8B). Morphometric measurements of the alveolar air space [6] calculated from 6 different images from each group (n = 3) indicate that the total area of the alveolar air space (28520 ± 1938) in the p-BSQ group increased significantly (p < 0.001) from that (16756 ± 1138) of the saline group, while the surface density {S/V (surface per unit volume), 0.1262 ± 0.0189} in the p-BSQ group decreased significantly (p < 0.001) from that of the saline group (S/V = 0.1935 ± 0.0201). Administration of vitamin C (15 mg/guinea pig/day) in the p-BSQ-treated animals prevented apoptosis to about 80% and lung damage about 85%.

Like that observed after CS exposure, intratracheal instillation of p-BSQ in the lungs of vitamin C-restricted guinea pigs also produced inflammation as evidenced by marked increase in the number of neutrophils in BALF. In separate experiments with guinea pigs (600–700 g, n = 3) deprived of vitamin C for 7 days, we observed that instillation of 300 μg of p-BSQ in 500 μl of normal saline for two successive days resulted in significant increase (p < 0.05, compared to sham controls) in both the total number of leukocytes (220 ± 25 × 10^4^) and the neutrophils (176 ± 20 × 10^4^) in the BALF. The corresponding values from sham controls that received only 500 μl of saline were leukocytes, 164 ± 12 × 10^4^, neutrophils, 20 ± 3 × 10^4^.

## Discussion

Emphysematous lung damage is a prominent component of COPD, which is a major and increasing global cause of mortality and morbidity. It is projected to become the 3^rd ^commonest cause of death and 5^th ^commonest cause of disability in the world by the year 2020 [40,41]. Cigarette smoking is by far the commonest cause of emphysema in Westernized countries, accounting for about 95% of cases [42]. However the cellular and molecular mechanisms of cigarette smoke-induced lung damage are not yet clear. Numerous studies investigating the role of smoking in the pathogenesis of emphysema have been carried out in chronic smokers. The drawback of studying the effects of actual smoke exposure is likely the effect of already developed lesions in the lungs on the response to smoke [43]. In order to understand the mechanism of initiation and sequential progress of the lesions as well as development of therapeutic approaches, researchers have produced various experimental animal models, including exposure to CS [41]. Earlier it had been hypothesized that several events, including apoptosis of lung cells, oxidative stress and proteolysis interact as means by which the lung is destroyed in emphysema [1]. But the sequential role of the events in typical pathology of emphysematous lung damage is still poorly understood. It had been suggested that a vicious cycle might be operating, because cells undergoing apoptosis display increased oxidative stress, which further contributes to the apoptosis [44]. Using a CS-induced guinea pig model, here we demonstrate that oxidative modification of lung proteins is the early event of CS-exposure. Apoptosis is a late event. Protein carbonyl is formed in the lung after exposure of the guinea pigs to smoke from only one cigarette, which is markedly increased after exposure to smoke from three cigarettes. No apoptosis is noticed during this period.

Considering that the main risk factor for emphysematous lung damage is chronic smoking, it has been suggested that study should be made to understand the response to the first smoke exposure of naïve lung to assess the relevant changes that may have a role in the first step of emphysema development [22]. It has also been suggested that an acute smoking model can give clear and more specific information about the cellular and molecular mechanisms of CS-induced lung damage [22]. Using histopathlogy and morphometric measurements here we show that while oxidative damage is the initial event, which is observed even after first smoke exposure, significant lung damage occurs on and from the 7^th ^day of smoke exposure up to 21^st ^day of the experimental period. As done before [6], the extent of lung damage has been evidenced by increased alveolar air space and significant decrease (p < 0.05) of the surface density (S/V) of the air space. Lung destruction in the CS-exposed guinea pigs has also been evidenced by significant increase in the mean linear intercept (Lm) and destructive index (DI) of the lung sections. As reported before, we have used vitamin C-restricted guinea pigs to minimize the vitamin C level in the tissues [6]. This is because vitamin C is a potential inhibitor of CS-induced oxidative damage [7]. Once oxidative damage is prevented by vitamin C, the subsequent physiological events, including lung damage, is prevented. Reports from other laboratories indicated that that it took at least four months of CS exposure to get appreciable lung damage in guinea pigs [13]. One reason might be that those workers did not restrict vitamin C in the diet of the animals. This would also explain why it is difficult to produce CS-induced lung damage in rats [45]. Rats not only synthesize vitamin C but also various drugs possessing completely unrelated chemical and pharmacological properties, including carcinogens, stimulate markedly the biosynthesis of vitamin C in rats [46-48].

A number of reports indicate that apoptosis plays a crucial role in CS-induced lung damage [1,2,6,34,44,58]. It has been proposed that cells undergoing apoptosis display increased oxidative damage, which further contributes to the apoptosis [44]. Here we show that oxidative protein damage is followed by apoptosis, which is accompanied by lung damage. Apoptosis is a tightly regulated mechanism of cell death, a process by which the cells eliminate unwanted damaged cells. The markers of apoptosis include DNA fragmentation, activation of caspase 3 and over expression of the proapoptotic Bax [49-53]. Caspases are aspartate-directed cysteine proteases with a pivotal role in apoptosis. Caspase 3 is an important caspase in the execution of downstream events in apoptosis. To evaluate apoptosis in human emphysema, Imai et al. [53] analyzed lung tissue of emphysema patients and individuals without emphysema. DNA fragmentation (TUNEL) revealed higher apoptosis in emphysematous than normal lung. Those authors also detected an increase in active caspase-3 and proteolytic fragment of PARP (a substrate of active caspase 3) in emphysematous lung. Moreover, expression of the proapoptotic protein Bax, but not the antiapoptotic protein Bcl-2, was detected in emphysematous lung tissue. It is known that the ratio of Bax and Bcl-2 determines whether a cell will undergo apoptotic death or not. In our guinea pig model of CS-induced lung damage, here we demonstrate a marked increase in DNA fragmentation (increase in TUNEL positive cells), activation of caspase 3 and cleavage of PARP. There was a rise in the level of the proapoptotic Bax protein, but no rise in the level of the antiapoptotic Bcl-2, resulting in an increase of the Bax/Bcl-2 ratio. This would indicate that there is a similarity of lung damage in our CS-induced guinea pig model with that of human emphysema.

Previously we had indicated that CS causes oxidative damage to guinea pig lung proteins both *in vitro *and *in vivo *that was prevented by vitamin C [7,8]. Here we show that administration of a moderately large dose of vitamin C (15 mg/guinea pig/day) not only prevents the oxidative protein damage, but also apoptosis and lung lesions. However, once the lung is damaged, neither administration of vitamin C nor discontinuation of the smoke exposure reverses the lung injury. Vitamin C prevents CS-induced protein oxidation and thereby prevents the subsequent events of apoptosis and lung damage. In CS-exposed guinea pigs, a moderately large dose of vitamin C (15 mg/day) is needed in order to maintain adequate tissue level of vitamin C. This is because CS consumes vitamin C [7] and a maintenance dose of 1 mg/day or 5 mg/day is inadequate to sustain sufficient tissue vitamin C content after smoke exposure.

Chronic exposure to CS in a rodent model causes inhibition in VEGF and VEGFR2 and it has been proposed that inhibitions of the growth factor and growth factor receptor lead to oxidative stress and apoptosis, which in turn result in emphysematous lung damage [31-34,44]. In our CS-induced guinea pig model we show that oxidative protein damage but not decreases in the expressions of VEGF and VEGFR are the initial events. However, it is possible that experimental emphysema caused by VEGFR blockade may involve a mutual feedback interaction between apoptosis and oxidative stress [44].

Continental European studies have shown that NAC reduces the number of exacerbation days in patient with COPD [14,15]. However; this was not confirmed in British Thoracic study of NAC [13]. A recent study showed that administration of NAC to CS-exposed mice did not decrease emphysema severity [54]. Here we show that while vitamin C almost completely prevents CS-induced oxidative protein damage, apoptosis and lung injury in the guinea pig, NAC is only partially effective.

The results obtained on the effect of CS exposure in the lung tissue *in vivo *have been corroborated by studies *ex vivo *with lung epithelial cells (A549). CS is a complex mixture of about 4000 compounds and it is yet to be known how many of them are responsible for oxidative damage, apoptosis and lung injury. Studies made by Ramage et al. had indicated that tobacco smoke initiates apoptosis in A549 airway epithelial cells as a result of mitochondrial damage and that this results in full apoptosis [55]. These authors had also shown that nicotine or aldehyde present in CS are not responsible for apoptosis and it had been suggested that some stable molecule(s) present in CS may initiate such apoptosis [55]. We had previously shown that some stable compound(s) present in aqueous extract of CS (AECS) induces protein oxidation in the lung tissue [7]. We considered that the stable compound might be p-BSQ, a long-lived radical. Although the presence of semiquinones in AECS was indicated in other laboratories [5], p-BSQ was not isolated and its biological effects were not separately studied. Rather the effects of semiquinones *in vitro *were shown in a mixture containing other components of aqueous extract of AECS [5]. We have isolated p-BSQ from AECS and characterized it separately. In this manuscript we have presented data to show that p-BSQ mimics AECS in inducing protein oxidation, proteolysis and apoptosis both *in vitro *and in A549 epithelial cells. p-BSQ also mimics CS-induced inflammation, apoptosis and lung damage *in vivo*. Tracing to the mechanism of action of p-BSQ, we have demonstrated that all the effects of p-BSQ, namely, protein oxidation, production of protein-Michael adduct, proteolysis and apoptosis occur via the formation of p-BQ.

Over the last few decades, inflammation and a protease/antiprotease imbalance have been proposed to act as downstream effectors of the lung destruction following chronic cigarette smoking, which accounts for 95% of cases of emphysema [1]. However, the emphasis on alveolar matrix destruction by the combination of inflammation and excessive proteolysis has failed to fully account for the mechanisms behind the eradication of septal structures [56]. Since apoptosis is a hallmark of emphysematous lung injury, researchers have addressed the question whether proteases can directly induce alveolar apoptosis and trigger emphysema. It was observed that intratracheal instillation of elastase produced emphysema in rats [57]. Aoshiba et al. [58] showed that intrabronchial delivery of active caspase 3 or nodularin (a serine/threonine kinase inhibitor) caused alveolar apoptosis and emphysema in rats. However, it would appear that the mechanism of apoptosis and emphysematous lung damage caused by intrabronchial instillation of proteases has less relevance to lung damage induced by cigarette smoke. Until now there is no report of direct injury to alveolar cells produced by any major component of cigarette smoke. Here we have shown that intratracheal instillation of p-BSQ, a major long-lived radical of CS, caused apoptosis and lung injury in guinea pigs. The results are comparable to that observed in the guinea pig lungs after direct exposure to whole CS.

Taken together, all the aforesaid results suggest that p-BSQ may be a major cause for producing CS-induced emphysematous lung damage. The mechanism of CS-induced lung damage induced by AECS is mimicked by p-BSQ. The sequence of events in both the cases is oxidative protein damage, proteolysis, apoptosis, ultimately leading to lung injury. *In vitro *and *ex vivo *results in lung epithelial cells show that p-BSQ induces all the pathophysiological conditions via the formation of p-BQ. In the presence of transition metal containing proteins, p-BSQ is rapidly converted to p-BQ. It would thus appear that *in vivo *in the lungs of smokers, p-BSQ of CS might damage the lungs via formation of p-BQ. Vitamin C reduces and inactivates p-BQ and thereby prevents the lung injury as shown in Scheme (9).

**Figure 9:**
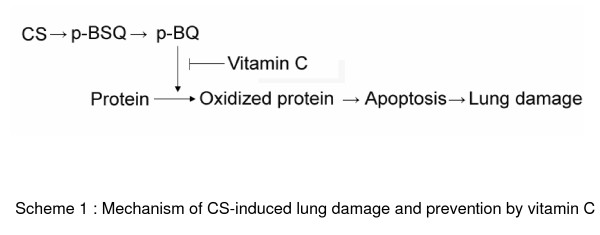
**Mechanism of CS-induced lung damage and prevention by vitamin C.**p-BSQ of CS induces all the pathological conditions via the formation of p-BQ. Vitamin C reduces and inactivates p-BQ and thereby prevents the lung injury

In this paper, using *in vitro*, *ex vivo *and *in vivo *models, we have attempted to provide answers to hypotheses regarding the mechanism of CS-induced lung damage. The mechanism of lung damage induced by AECS is similar to that caused by p-BSQ, a major long-lived radical of CS. But these models have both advantages and limitations. There are similarities in the mechanism of lung injury presented in our guinea pig model with that of human emphysema. However, one major point should be taken into consideration before comparing our results obtained in guinea pigs with that of humans. While all the guinea pigs exposed to CS had emphysematous lesions, only about 15% of smokers develop emphysema [59]. So, some additional factors like genetic predisposition and the nutritional status of the subject, particularly the vitamin C status, might also be critical in determining the susceptibility of a smoker to emphysema. Nevertheless, since there is no novel or even currently effective treatment aimed at this irreversible fatal disease [60], a practical approach would be prevention of emphysema. Undoubtedly the best way of prevention is to stop smoking. However, approaches to cessation of smoking have had limited success. So, if the results obtained with guinea pigs are applicable to humans, intake of moderately large doses (about 2 g/day) of vitamin C may protect the smokers from emphysematous lung damage.

## Abbreviations

CS: cigarette smoke; AECS: aqueous extract of cigarette smoke; p-BSQ: p-benzosemiquinone; p-BQ: p-benzoquinone; NAC: N-acetylcysteine; VEGF: vascular endothelial growth factor; VEGFR2: vascular endothelial growth factor receptor 2; BALF: broncho-alveolar lavage fluid; vit C: vitamin C; TRITC: tetramethyl Rhodamine iso-thiocyanate; CC3: cleaved caspase 3.

## Competing interests

The author(s) declare that they have no competing interests.

## Authors' contributions

SB carried out major part of the experiments on cell biology and cell signalling. RC did the major work on the purification and characterization of p-BSQ. IBC planned and also designed some of the experiments as well as wrote the manuscript, including revision. AG did some experiments on immunoblotting. HK carried out intratracheal instillation of p-BSQ. SR collaborated in physico-chemical analyses of p-BSQ. KP and DJ participated in the study of oxidative damage. KP also edited the Figures. All authors read and approved the final manuscript.

## Supplementary Material

Additional file 1 **Identification and characterization of p-benzosemiquinone (p-BSQ).** p-BSQ was identified by HPLC and characterized by various physico-chemical analyses, including UV, mass, NMR and ESR spectroscopy. The file contains legends of additional figure 1 (see additional file 2) and legends of additional figure 2 (see additional file 3). Click here for file

Additional file 2 **Additional figure 1 depicting HPLC, mass, UV, NMR and ESR spectra.** Click here for file

Additional file 3 **Additional figure 2 depicting mass spectra of p-BQ-BSA covalent Michael adduct.** We performed MALDI-TOF-MS analyses of BSA before and after incubation with p-BSQ or p-BQ at pH 7.4. We observed that incubation of 100 μg of BSA (MW 66,340 Da) with 185 nmoles of p-BQ produced an adduct of MW 68,347 Da, indicating association of 19 nmoles of p-BQ in the BSA molecule. When p-BQ was replaced by p-BSQ, the MW of the product was found to be 67,292 DA, indicating the addition of 9 nmoles of p-BQ. Click here for file
