# Supplementary material for: Cellular and molecular mechanisms of cigarette smoke-induced lung damage and prevention by vitamin C
Source: J Inflamm (Lond). 2008 Nov 11;5:21. doi: 10.1186/1476-9255-5-21 (PMC2615750; doi:10.1186/1476-9255-5-21)
Supplement: Additional file 1 — Identification and characterization of p-benzosemiquinone (p-BSQ). p-BSQ was identified by HPLC and characterized by various physico-chemical analyses, including UV, mass, NMR and ESR spectroscopy. The file contains legends of additional figure 1 (see additional file 2) and legends of additional figure 2 (see additional file 3). [file 1476-9255-5-21-S1.doc]

***Additional file 1***

***Identification and Characterization of para-benzosemiquinone (p-BSQ)***

p-BSQ has been identified and quantified by HPLC (see *Additional* Fig. 1 A). The structure of p-BSQ has been determined by mass spectrometry (molecular mass of 109 Da, Fig. 1 B), UV (Fig. 1 C), ^13^C-NMR (Figs not shown), ^1^H-NMR (Figs. 1 D, E) and ESR spectroscopy (Fig. 1, F). UV-spectroscopy shows that λmax of p-BSQ in water is 288 nm (ε_288_ = 6976 M^-1^ cm^-1^). The ^1^H-NMR profile in CD_3_COCD_3_ (Fig. 1D) shows one sharp peak at 6.56 ppm corresponding to the four aromatic protons of hydroquinone structure and another peak at 7.55 ppm corresponding to the heterolinked proton (s), the proton (s) linked to the oxygen atoms in hydroquinone (Figs. 1 D, E). The figure (Fig. 1D) further shows that the ratio of the aromatic protons to the heteroatomic protons in p-BSQ is 1: 0.2589 (approximately 4:1), which is nearly half of that in HQ (1: 0.4143, approximately 4:2, Fig. 1 E). p-BSQ has been finally identified by ESR spectroscopy of the freshly prepared compound. The ESR spectrum (Fig. 1 F) shows the presence of a single symmetrical line, indicative of a single type of radical consisting of an unpaired electron delocalized over an aromatic framework. The g-value, calculated with reference to diphenyl picryl hydrazyl radical, has been found to be 2.004680, which is practically identical with the g-value of p-BSQ (2.004679), as reported before [Wertz JE, Bolton JR: **Electron spin Resonance, Elementary theory and Practice Applications**. McGraw-Hill Book Company, New York 1972, pp 313 and 465.]

**Legends of *Additional* Figure 1**

**A,** HPLC profiles of p-BSQ; a, b HPLC profiles using silica column (normal phase) and c, d that using ODS column (reverse phase); a, c represent p-BSQ in AECS and b, d represent pure p-BSQ isolated from AECS. Twenty μl of 40 times diluted AECS or 100 ng of purified p-BSQ was injected in the HPLC. HPLC was carried out using a Shimadzu 10A VP instrument attached to a UV detector and a chromatopac C-R6A. The column used was a normal phase silica column (Merck, LiChrospher® Si 60, 25 cm, 5 µm). The mobile solvent was methylene chloride: methanol (90:10,v/v) and the flow rate, 0.5 ml/min. p-BSQ was detected at 293 nm at retention time (t_r_), 8.808 min

( *Additional* Fig.1 A, b) and that in AECS, 8.813 min (*Additional* Fig1 A, a). HPLC was also carried out using a reverse phase Shimadzu Shim-pack CLC-ODS (M) column (25 cm, 5 µm), where the mobile solvent was water: methanol (95:5, v/v) and the flow rate, 0.8 ml/min. p-BSQ was detected at 288 nm at t_r_, 13.458 min (*Additional* Fig. 1 A, d) and that in AECS, 13.467 min (*Additional* Fig.1 A, c).

**B,** Mass spectrum was taken in a JEOL JMS600 mass spectrometer using ionization mode ESI +ve ion mode.

**C,** UV spectrum of p-BSQ (5 μg) in water taken in a Hitachi spectrophotometer (model U3020).

**D,** ^1^H-NMR spectroscopic profile of HQ (200µg) in CD_3_COCD_3_ (500µl) and **E** ^1^H-NMR spectroscopic profile of p-BSQ (200µg) in CD_3_COCD_3_ (500µl) taken in a Bruker 500MHz spectrophotometer.

E, ESR spectrum of p-BSQ isolated from freshly prepared AECS from 100 cigarettes. The spectrum was recorded in a JES-REIX ESR spectrometer (Tokyo,Japan). The spectral parameters were: microwave frequency, 9.435 GHz; power, 2 mW; field modulation width, 0.4 mT; modulated frequency, 100 kHz; time constant, 0.3 sec; scan rate, 2.5 mT/sec; temperature, 25^0^ C; g value was calculated to be 2.004680 .

Legends of *Additional* Figure 2

p-BSQ / p-BQ-induced protein (BSA) modification as evidenced by Mass spectra. **A**, mass spectra of BSA; **B**, mas spectra of BSA-p-BQ adduct; **C, mass spectra of BSA-p-BSQ** adduct. 100 µg of BSA was incubated with 180 nmoles of p-BSQ or p-BQ in 200 µl of 10 mM ammonium bicarbonate buffer pH 7.4, at 37^0^C for 2 h. After incubation the proteins were dialysed against 10 mM ammonium bicarbonate buffer for 12 hrs to free of excess p-BSQ/ p-BQ, lyophilized and then subjected to MALDI –TOF-MS (Applied Biosystems 4700 Proteomics Analyzer 347000152).
