# Supplementary figures and images for: Cellular and molecular mechanisms of cigarette smoke-induced lung damage and prevention by vitamin C
Source: J Inflamm (Lond). 2008 Nov 11;5:21. doi: 10.1186/1476-9255-5-21 (PMC2615750; doi:10.1186/1476-9255-5-21)

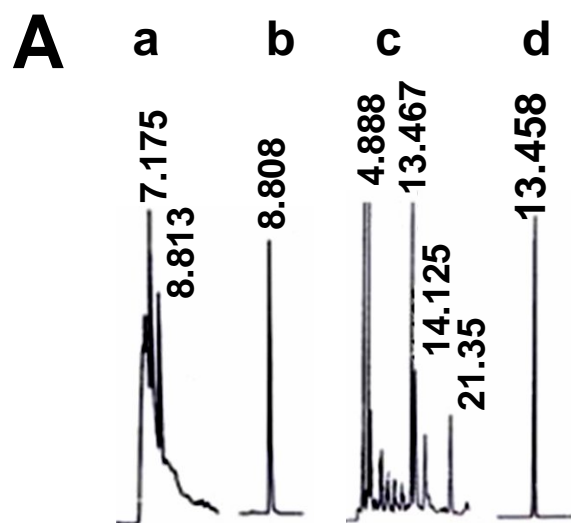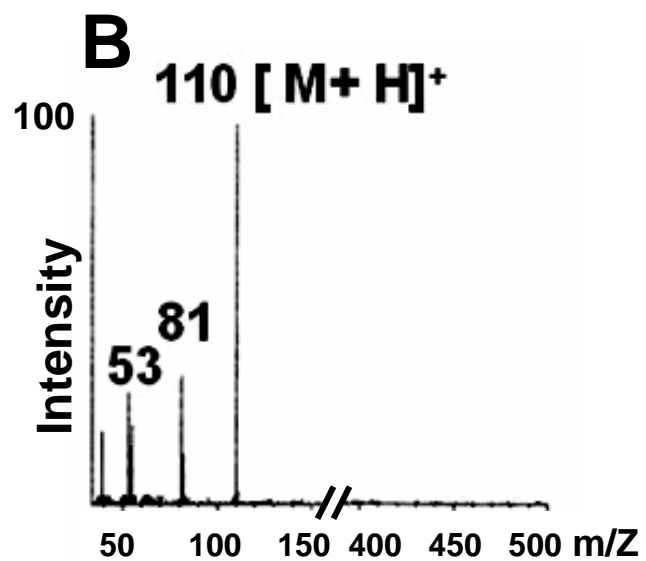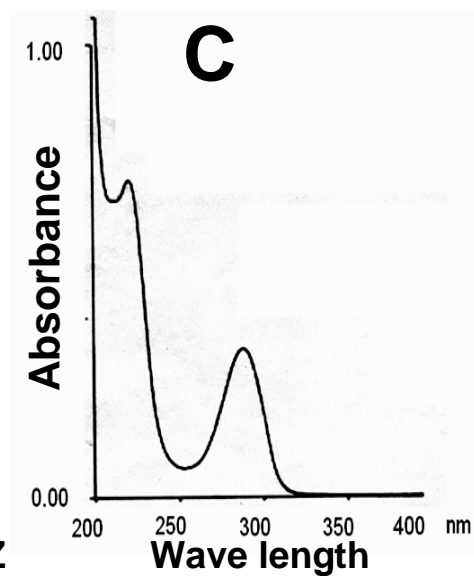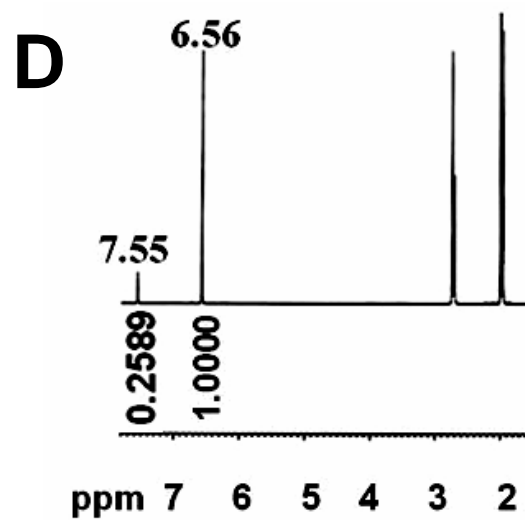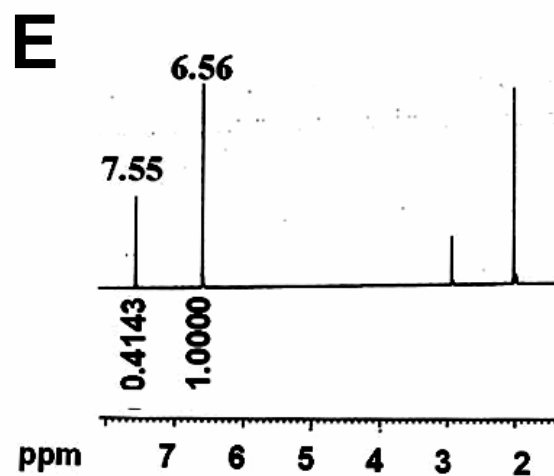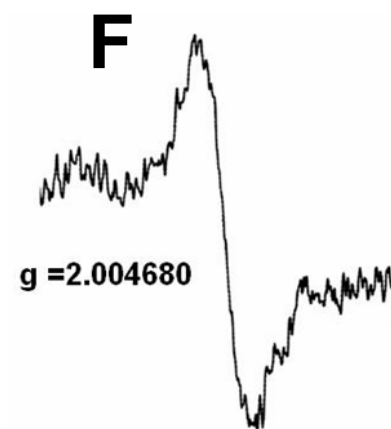

Supplement: Additional file 2 — Additional figure 1 depicting HPLC, mass, UV, NMR and ESR spectra. [file 1476-9255-5-21-S2.pdf]

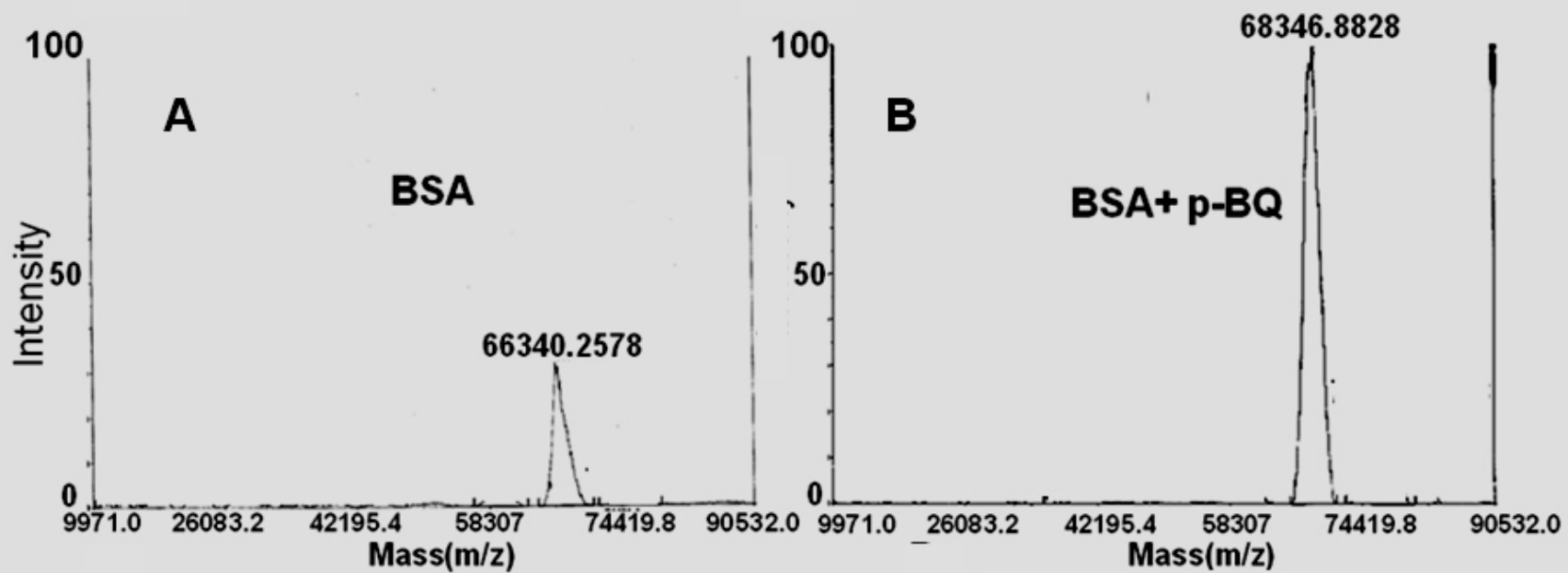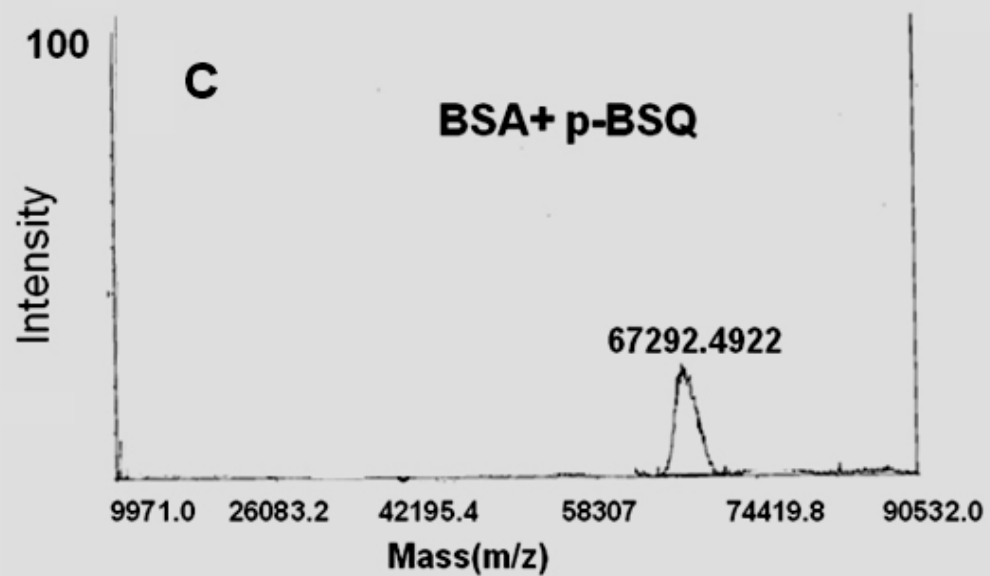

Supplement: Additional file 3 — Additional figure 2 depicting mass spectra of p-BQ-BSA covalent Michael adduct. We performed MALDI-TOF-MS analyses of BSA before and after incubation with p-BSQ or p-BQ at pH 7.4. We observed that incubation of 100 μg of BSA (MW 66,340 Da) with 185 nmoles of p-BQ produced an adduct of MW 68,347 Da, indicating association of 19 nmoles of p-BQ in the BSA molecule. When p-BQ was replaced by p-BSQ, the MW of the product was found to be 67,292 DA, indicating the addition of 9 nmoles of p-BQ. [file 1476-9255-5-21-S3.pdf]
